# In Vitro Evaluation of Virucidal Effect of Polysaccharides Extracted and Purified from *Arthrospira platensis* and *Dunaliella salina* on Human Adenovirus Type 5 in A549 Cells

**DOI:** 10.3390/molecules31061023

**Published:** 2026-03-19

**Authors:** Marco Verani, Clementina Manera, Alessandra Pagani, Matteo Banti, Annalaura Carducci, Federica Gasperin, Alice Cannaos, Graziano Di Giuseppe, Lionella Palego, Paola Nieri, Ileana Federigi

**Affiliations:** 1 Hygiene and Environmental Virology Laboratory, Department of Biology, University of Pisa, Via S. Zeno 35/39, 56123 Pisa, Italy; marco.verani@unipi.it (M.V.); annalaura.carducci@unipi.it (A.C.); f.gasperin@studenti.unipi.it (F.G.); a.cannaos@studenti.unipi.it (A.C.); ileana.federigi@unipi.it (I.F.); 2 Interdepartmental Center of Marine Pharmacology (MarinePHARMA), University of Pisa, 56126 Pisa, Italy; clementina.manera@unipi.it (C.M.); graziano.di.giuseppe@unipi.it (G.D.G.); paola.nieri@unipi.it (P.N.); 3 Department of Pharmacy, University of Pisa, Via Bonanno 6, 56126 Pisa, Italy; matteo.banti@phd.unipi.it; 4 Zoology and Anthropology Unit, Department of Biology, University of Pisa, Via A. Volta 4, 56126 Pisa, Italy; 5 Department of Clinical and Experimental Medicine, Via Savi 10, 56126 Pisa, Italy; lionella.palego@unipi.it

**Keywords:** virucidal activity, *Arthrospira platensis*, *Dunaliella salina*, human adenovirus type 5, polysaccharides, A549 cell line, virucidal assay, non-enveloped virus

## Abstract

Polysaccharides derived from cyanobacteria and microalgae have attracted increasing interest as natural virucidal agents. Among them, polysaccharides from the cyanobacterium *Arthrospira platensis* (*A. platensis*), and the green microalgae *Dunaliella salina* (*D. salina*) have shown virucidal activities, mainly against enveloped viruses, while evidence on non-enveloped viruses is still limited. In this study, the virucidal activity of purified polysaccharides extracted from *A. platensis* (APPs) and from *D. salina* (DSPs) was evaluated in vitro against human adenovirus type 5 (HAdV5), a non-enveloped pathogenic virus with high persistence in the environment and resistance to disinfection. The in vitro assays were carried out at concentrations previously verified as non-toxic by morphological evaluation of A549 cells after 24 and 48 h of incubation, testing two viral loads, namely, 10^3^ and 10^4^ tissue culture infectious dose 50% per milliliter (TCID_50_/mL). For APPs, a possible time-dependent effect was also assessed at different contact times (15, 30 and 60 min). DSPs showed a limited virucidal effect related to the starting viral concentration, while APPs induced a consistent viral reduction (up to 98.8%) at both viral concentrations. The virucidal effect of APPs occurred rapidly and was not significantly influenced by contact time, thus suggesting that prolonged exposure is not a determining factor for polysaccharide virucidal activity. These findings demonstrate the virucidal activity of APPs against a highly resistant non-enveloped virus and provide preliminary in vitro evidence of their potential application as natural virucidal agents, particularly for environmental disinfection purposes. Further investigations are warranted to elucidate the underlying mechanisms of action and to optimize their practical use.

## 1. Introduction

The spread of human pathogenic viruses by direct and indirect transmission represents a major public health concern, especially in the context of emerging and re-emerging infectious diseases. Thus, the investigation of natural compounds for their effects on viral viability has gained increasing attention, especially with regard to their potential application in environmental matrices. In fact, the use of naturally derived virucidal and antiviral agents may represent a more sustainable alternative to conventional synthetic disinfectants, where virucidal agents directly inactivate virus particles and antiviral agents inhibit viral replication within host cells [1]. Human adenovirus (HAdV) represents a valuable candidate for testing the effect of disinfectant products given its clinical relevance and its structural properties. First identified in 1953 from surgically removed adenoids [2], HAdV are currently classified into seven species (from A to G), comprising more than 100 different types associated with a wide range of clinical manifestations, from respiratory infections (species B, C, E) to conjunctivitis and keratoconjunctivitis (mainly species C and D) and gastrointestinal problems (mainly species F) [3,4]. Given their fecal excretion, HAdV types are frequently detected in wastewater [5], and their ability to persist in environmental matrices has supported the use of HAdV as an indicator of water fecal contamination [6,7] and of viral removal efficiency in wastewater treatment processes [8,9]. In fact, from a structural point of view, HAdV exhibits some key features that allow long-term persistence in the environment, such as the absence of an envelope and the stability of its DNA double-stranded genome [5]. For these reasons, HAdV often has been used to assess the virucidal efficacy of synthetic chemical products [10,11] and natural compounds intended for environmental applications [12,13].

Among natural sources of virucidal compounds, cyanobacteria and microalgae have attracted considerable interest due to their intracellular bioactive polysaccharides and exopolysaccharides. *Arthrospira platensis* Gomont (*A*. *platensis*) (synonym *Arthrospira fusiformis*, formerly *Spirulina platensis*, commonly known as Spirulina) is a Gram-negative filamentous bacterium mostly found in alkaline salt lakes; it is characterized by a rapid growth [14] and a high polysaccharide content, accounting for approximately 15–20% of its biomass [15]. *Dunaliella salina (Dunal)* Teodoresco (*D*. *salina*) is a unicellular, biflagellate green microalga adapted to survive in hypersaline environments (e.g., salt lakes and salt evaporation ponds), where it contributes to their pink color [16], and it is known to produce exopolysaccharides [17]. The polysaccharides derived from both organisms have been associated with several biological activities, including antioxidants, anti-inflammatory, immune-regulatory, antimicrobial and antiviral effects [18,19,20,21,22,23,24,25,26]. Evidence of virucidal and antiviral activity has been reported particularly for sulfated algal polysaccharides [27,28,29,30], including calcium-spirulan isolated from *A. platensis* [31], but such activity has been mainly described against enveloped viruses. It has been suggested that the negative charge of sulfate groups can interact with positively charged domains on the viral surface, thus preventing virus adsorption to host cells [32,33]. However, antiviral effects have also been described for neutral polysaccharides, indicating that inhibition of viral infection is not exclusively dependent on electrostatic interactions, but also on other molecular characteristics [34].

In this context, the present study aimed to address the limited data on the activity of microalgal-derived polysaccharides against non-enveloped viruses, focusing in particular on their virucidal effects, as these viruses are generally more resistant to inactivation. To this end, a preliminary evaluation was conducted to assess the effect of intracellular polysaccharides extracted from *A. platensis* (APPs) and exopolysaccharides from *D. salina* (DSPs) against HAdV5. The virucidal activity was assessed through in vitro experiments, including a comparison between APPs and DPSs and a kinetic evaluation of viral inactivation of APPs based on different contact times.

## 2. Results

### 2.1. Results of APPs and DSPs Effect on A549 Cell Lines

**Figure 1:**
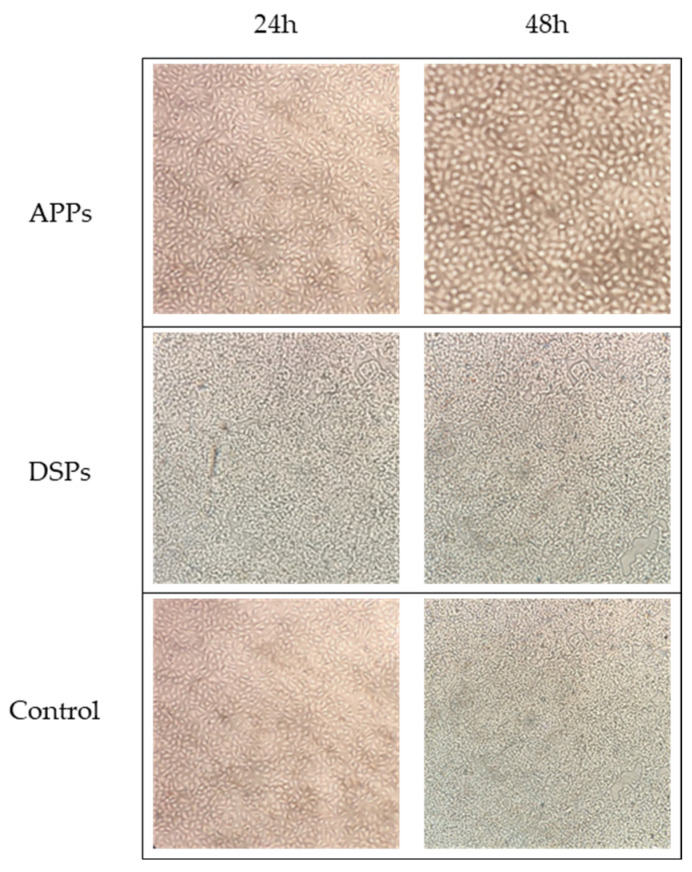
Morphological observation of A549 cells at 24 and 48 h following incubation with polysaccharides, compared to control. Images were acquired using an inverted light microscope at 100× magnification.

No evident morphological alterations were observed in A549 cells after 24 and 48 h of incubation with APPs (84.38 µg/mL) or DSPs (75.0 µg/mL), as assessed by microscopic examination compared with the control (Figure 1).

As morphological observation alone does not fully replace quantitative viability assays, cytotoxicity of APPs on A549 cells was previously determined using the 3-(4,5-dimethylthiazol-2-yl)-2,5-diphenyltetrazolium bromide (MTT) assay [35]. In that study, a concentration of APPs of approximately 250 µg/mL reduced cell viability by 50%, corresponding to the half-maximal inhibitory concentration (IC_50_), and concentrations up to 150 µg/mL did not significantly compromise cell viability. The APPs concentration employed in the present study (84.38 µg/mL) was, therefore, well below the previously established cytotoxic threshold, supporting the absence of direct toxic effects on the cell monolayer. For DSPs, no virucidal activity was detected under the experimental conditions tested (see Section 2.2); therefore, additional quantitative cytotoxicity assays (e.g., MTT) were not performed for this fraction.

Importantly, A549 cells pre-exposed to APPs or DSPs at the tested non-cytotoxic concentrations remained fully permissive to HAdV5 infection, showing viral titers of 10^4^ TCID_50_/mL, comparable to those observed in the cell monolayer infected with a stock solution of HAdV5 in the absence of polysaccharides (see Section 4.3. and Section 4.4).

This finding suggests that, under the adopted experimental conditions, the polysaccharides did not alter cellular permissiveness to HAdV5 infection.

### 2.2. Results of APPs and DSPs Effect on HAdV5

Viral infectivity was quantified as tissue culture infectious dose 50% per milliliter (TCID_50_/mL). Each virucidal assay was performed in triplicate, using two HAdV stock solutions at known viral concentration of 10^4^ and 10^3^ TCID_50_/mL (initial viral titers). After 30 min of contact with the polysaccharides, the residual viral infectivity (final viral titer) was determined, and results were expressed as geometric mean ± standard deviation (Table 1). Then, viral abatement was calculated considering initial and final viral titers, and expressed either as logarithmic or percentage reductions. The tested concentration of the APPs solution exhibited the same percentage reduction (98.78% ± 0.19) at both initial viral titers. In contrast, the DSPs solution showed no reduction at the higher initial viral titer (5.65 × 10^4^) but demonstrated a percentage reduction of 92.50% at the lower viral titer (5.65 × 10^3^) (Table 1). The negative control (see Section 4.6), as expected, showed the presence of the virus without any reduction in viral titer.

### 2.3. Results of APPs on HAdV5 at Different Contact Times

**Figure 2:**
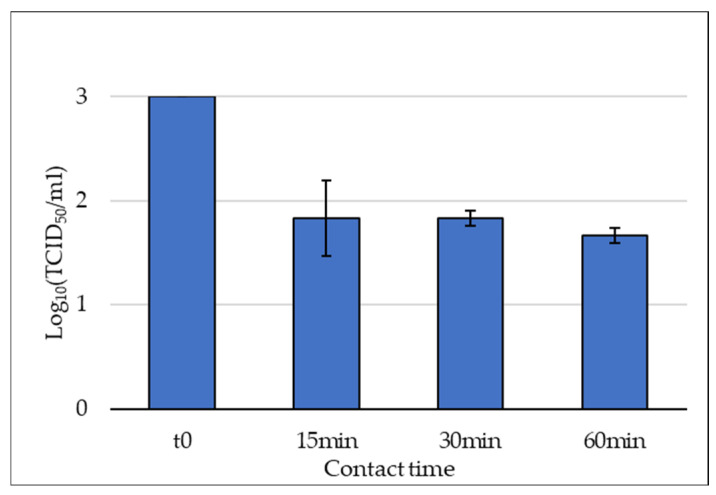
Viral titers of HAdV5 after exposure to APPs at 15, 30, and 60 min of contact time (log_10_-transformed). T0 represents the initial viral titer before exposure to the polysaccharides and was used as control. No significant differences were observed among time points (*p* = 0.5822).

In Table 2, the HAdV titers are reported for each trial along with their viral abatements, which were obtained by considering the titers of the replicates and the initial viral titer (4.24 × 10^3^ TCID_50_/mL). The percentage reductions varied from 91.21% at 15 min to 95.25% at 60 min. A slight increase in viral abatement was observed over time (Figure 2); however, this trend was modest and was not associated with statistically significant differences among the three contact times (one-way ANOVA, *p* = 0.5822), indicating that extending exposure from 15 to 60 min did not result in a significant additional reduction in viral infectivity. These findings suggest that the virucidal effect of APPs occurs rapidly and reaches a plateau within the first minutes of contact under the experimental conditions tested.

## 3. Discussion

In the last decade, interest in natural polysaccharides has increased considerably due to their potential applications in various fields, including their effect on human pathogens. As an example, marine-derived sulfated polysaccharides have been evaluated against a broad range of enveloped and non-enveloped viruses, demonstrating activity at different stages of the viral life cycle. Their effects include direct inactivation of viral particles prior to cell infection (virucidal activity) as well as inhibition of viral replication within host cells (antiviral activity) [36,37]. In the present study, we specifically investigated the virucidal activity of microalgal-derived polysaccharides characterized by different sulfate contents (APPs from *A. platensis* and DSPs from *D. salina*, with higher and lower sulfate levels, respectively) against HAdV5, a non-enveloped human virus known for its marked environmental persistence and resistance to disinfection.

APPs used for this work were previously described by Banti et al. [38]. In that study, APPs (formerly named SPPs) showed protective effect against cisplatin toxicity on House Ear Institute-Organ of Corti (HEI-OC1) cells, inducing a significant increase in cell viability at 40 μg/mL and 80 μg/mL and inhibiting, at 80 μg/mL, the cisplatin-induced increase in ROS level. More recently, Polini et al. [35] reported that the same APPs exerted in vitro selective cytotoxic effects against A549 lung cancer cells (IC_50_ estimated at approximately 250 μg/mL) by promoting redox imbalance, apoptosis, and immune response, without affecting the viability of non-cancerous bronchial epithelial cells (16HBE). In the present work, APPs tested against HAdV at 84.38 µg/mL exhibited a 98.78% reduction at both starting viral concentrations, with viral abatement of approximately 91.21% observed within the first 15 min. Similarly, Chen et al. [39] showed that polysaccharides from *A. platensis* acting during the early stages of influenza A virus infection reduced the viral titer by approximately 90%. On the other hand, Chen et al. [40] demonstrated that the polysaccharides extracted from *A. platensis* (0.2 μg/mL) exhibited a reduction in respiratory syncytial virus (RSV) titer by 80%. However, few data are available on the effects of sulfated polysaccharides from *A. platensis* against non-enveloped viruses. A previous study by El-Baz et al. [41], using ethanol extract, reported moderate reductions in viral infectivity for adenoviruses, with percentages generally lower than those observed in the present study: 53.3% for Adenovirus type 7, 50% for Adenovirus type 40, 66.7% for Coxsackievirus B4, and 76.7% for Astrovirus type 1. However, most of the available literature on sulfated polysaccharides from *A. platensis* investigated antiviral, rather than virucidal, activity against enveloped viruses. As early as 1996, Hayashi et al. [31] demonstrated a notable antiviral effect of calcium spirulan (0.23 μg/mL) on several enveloped viruses, including human cytomegalovirus, measles virus, mumps virus, influenza A virus and HIV-1. Subsequent studies confirmed the ability of spirulina-derived polysaccharides to interfere with viral attachment and entry, as well as to reduce viral replication during the early stages of infection. For example, Mader et al. [42] reported a specific effect of calcium spirulan (0.04 μg/mL) on the attachment of Herpes Simplex Virus 1, with a reduction in infectivity of around 50%.

In contrast, polysaccharides obtained from *D. salina* exhibited a viral-load-dependent effect, with a detectable activity only at lower viral load. Specifically, DSPs tested at a concentration of 75 µg/mL did not induce a significant reduction in viral infectivity when incubated with a high viral titer (5.65 × 10^4^ particles/mL), whereas a reduction of 92.50% was observed at the lower viral concentration (5.65 × 10^3^ particles/mL). Similarly, Ohta et al. [43] showed that extracts from another *Dunaliella* species (*Dunaliella primolecta*) at different concentrations (20, 10, 5, 1, 0.5 µg/mL) did not exhibit virucidal activity on Adenovirus Type VI strain (Ad75), while being effective against Herpes Simplex virus type 1, an enveloped virus. These data, together with those obtained in our study, suggest that the limited efficacy of DSPs against adenoviruses could be related both to viral concentration in the matrix and to the viral structure (presence of envelope). Thus, these findings highlight the need to investigate higher concentrations of DSPs while carefully monitoring cytotoxicity. In this context, a structured dose–response study would be required to systematically evaluate virucidal efficacy across increasing concentrations and to define a potential therapeutic window, namely, the concentration range at which virucidal activity is achieved without inducing detectable cytotoxic effects.

Overall, the greater efficacy of APPs compared to DSPs observed in the present study is consistent with the findings of Singab et al. [44], which reported a higher viral abatement of polysaccharides from *A. platensis* compared to those from *D. salina*, with mean values of approximately 72% and 29%, respectively, against enveloped viruses such as Hepatitis C Virus. Nevertheless, the magnitude of viral reduction achieved by APPs in this study can still be considered weak to moderate and remains below the 2-log reduction generally required for hygienic practices (e.g., handwashing), as reported in the international standard BS EN 14476:2025 [45].

Although the exact mechanism of action was not investigated in this study, possible explanations include a direct interaction with viral particles leading to inhibition of adsorption [39] or interference with early stages of the viral replication cycle. Moreover, it could be useful to clarify the role of sulfated groups of APPs and DSPs in virucidal activity; for example, the lower number of sulfated polysaccharides present in DSPs compared to those present in APPs (1.98 ± 0.3% vs. 9.7 ± 0.9%, see Section 4.1. and Section 4.2) could contribute to further explaining this different efficacy. In fact, previous studies suggest that this effect may be mediated by interaction between negatively charged sulfate groups and positive charges domains on the viral surface, potentially interfering with the viral recognition processes [46]. Moreover, recent evidence suggests that sulfated polysaccharides from marine algae may also affect later stages of the viral replication cycle after viral internalization into host cells, as well as modulate and improve the host immune responses [36,37,47].

### Limitations

Some methodological limitations of the present study concern the design of the virucidal assay, in terms of the concentrations of polysaccharides tested and the procedure adopted to stop their activity before viral titration. First, the working solutions were selected based on the availability of purified material and on previously verified non-cytotoxic concentrations. However, the final tested concentrations were not identical, as APPs were evaluated at a slightly higher concentration than DSPs (84.38 µg/mL and 75.0 µg/mL, respectively). Although these concentrations are relatively close, DSPs were tested at a lower dose. Considering that virucidal activity is generally concentration-dependent, it cannot be entirely excluded that the lower activity observed for DSPs may have been partially influenced by the lower concentration tested. Therefore, the direct quantitative comparison between the two polysaccharide fractions should be interpreted with caution.

Second, in the virucidal assay, polysaccharide activity was interrupted by serial dilution of the virus–compound mixture prior to viral titration. Due to the structure of the experimental protocol, no additional neutralization step (e.g., chemical neutralizers, filtration, or compound removal procedures) was applied; thus, it cannot be completely excluded that a minor fraction of the observed viral reduction may have occurred during the early phase of the titration procedure.

Moreover, in this study we did not perform a reference test for virus inactivation using reference virucidal substances (e.g., formaldehyde, glutaraldehyde, and peracetic acid), as recommended by international standards on chemical disinfectants [45]. Therefore, the absence of standardized positive control does not allow verification of the stability of the spiking virus and of the performance of the experimental system.

## 4. Materials and Methods

### 4.1. APPs Preparation: Extraction and Purification

The extraction, purification and proximate composition analysis of APPs (formerly called SPPs) used for this work were previously described by Banti et al. [38]. Briefly, the biomass of *Spirulina platensis* was purchased from Spirufarm Srl (Casalbuttano ed Uniti, Cremona, Italy). The APPs were obtained by ultrasound-assisted extraction combined with hot water extraction, followed by deproteinization and decolorization treatment. The yield was 3.7%, with monosaccharide and sulfate group contents of 80.5 ± 2.1% and 9.7 ± 0.9%, respectively. The monosaccharide composition previously reported [38] indicates that APPs are mainly composed of glucose/galactose, together with rhamnose, ribose, and fucose.

### 4.2. DSPs Preparation: Extraction and Purification

The green alga *Dunaliella salina* was grown in sterilized artificial seawater enriched with Walne medium [48] in a 12 h light/dark regimen inside a daylight (Osram Daylight lamp, 36W/10; Osram, Garching near Münich, Germany) and fluorescent-illuminated incubator system (Osram Fluora lamp, 40W/77; Osram, Garching near Münich, Germany) at 20–22 °C. Centrifugation and filtration were used sequentially for the recovery of *D. salina* from the culture medium. The culture (1.0 × 10^9^ cells per liter) was centrifuged at 700× *g* for 2 min. After that, the supernatant was recovered and subjected to two levels of filtration, first using Sartorius cellulose nitrate filters (Prokeme SRL, Florence, Italy) with a pore size of 8 µm, and then the filtrate was filtered again using filters with a finer pore size of 0.45 µm. The clear supernatant was stored at 6 °C. The exopolysaccharides were obtained according to the method of Goyal et al. [23]. Briefly, 250 mL of filtrate was poured into a glass beaker and heated on a hot plate at 60–70 °C until the volume was reduced to one-fifth of the original. After cooling, cold methanol was added to reach a final volume of 300 mL, and the suspension was refrigerated at 4 °C for 18 h to precipitate the exopolysaccharides, which appeared as white floccules. The suspension was then centrifuged (10,000× *g* for 20 min), and the supernatant was discarded. The obtained pellet was suspended in 95% ethanol and centrifuged again (10,000× *g* for 10 min at 4 °C). This step was repeated twice. The pellet was then dried under nitrogen gas to obtain 360 mg of white powder, which was dissolved in 80 mL of MilliQ water. Dialysis was performed to remove salts against double-distilled water for 48 h, using membranes (Pur–A–Lyzer™ Mega Dialysis Kit, Sigma Aldrich, Milan, Italy) with molecular weight cut-offs (MWCO) of 1 kDa. The dialyzed solution was then treated with trichloroacetic acid (TCA, 20%) for 1 h to remove proteins. After that, it was centrifuged (48,000× *g* for 30 min at 4 °C) and the pellet of proteins was discarded. The supernatant was dialyzed again to remove TCA. The dialyzed solution was lyophilized to obtain 5.5 mg of DSPs as white powder, which was stored at 4 °C for biological evaluation. The phenol sulfuric assay [49] was carried out to assess the total carbohydrate content, which was 22.3 ± 2.5%. The sulfate group content, estimated using the previously described method [38], was found to be of 1.98 ± 0.3%.

### 4.3. Human Adenovirus Type 5 Preparation

HAdV5 was obtained from the American Type Culture collection (ATCC VR-5) (Manassas, VA, USA) and was propagated and assayed on the A549 cell line (ATCC CCL-185) (Manassas, VA, USA) as the host cell culture, according to the manufacturer’s instructions. Briefly, a small volume of viruses with 0.1 multiplicity of infection (MOI) was absorbed on 25-cm^2^ flasks for 1 h at 37 °C in a humidified 5% CO_2_ atmosphere. After adsorption, growth medium containing Minimal Essential Medium (MEM) (Biowest, Nuaillé, France) supplemented with L-glutamine (1%) (Biowest, Nuaillé, France), gentamicin (1.25‰) (Biowest, Nuaillé, France), and 2% fetal bovine serum (yourSIAL, S.I.A.L. Srl, Rome, Italy) was added, and the flasks were incubated for 2–3 days. Once the cytopathic effect (CPE) was detected by microscopic examination using an inverted light microscope, based on characteristic morphological changes (cell rounding, clumping and detachment from the monolayer), the flasks were subjected to three freeze–thaw cycles to induce virus release from cells [50]. Then, cell debris was pelleted with centrifugation at 160× *g* for 3 min, and the supernatant was collected to prepare 1 mL solutions at 10^4^ TCID_50_/mL, which were used as the stock solution for the virucidal assays (see Section 4.4 for viral quantification).

### 4.4. Viral Quantification

Viral quantification was performed by an endpoint dilution assay. The viral titer was measured using 96-well plates by seeding three ten-fold dilutions of each sample onto 8-well lines containing A549 cell culture. After five days of incubation at 37 °C in 5% CO_2_, the 50% endpoint of the inoculated cells was determined using the Spearman–Karber formula [51,52], as reported in Equation (1).

(1) $${Log}_{10}\left({TCID}_{50}\right)=-x_{0}+\frac{d}{2}-d\frac{\sum r_{i}}{n_{i}}$$

where TCID_50_ is the 50% tissue culture infective dose; x_0_ is the highest dilution giving 100% CPE; d is the log_10_ of the dilution factor (corresponding to 1, as samples were ten-fold diluted); r_i_ is the total number of test units showing CPE; n_i_ is the number of test units per dilution (8 in our study).

Then, the obtained titer of the viral suspension was adjusted according to the volume of the virus inoculum (75 µL) to express the results as TCID_50_ per milliliter (TCID_50_/mL).

### 4.5. Evaluation of APPs and DSPs Effect on A549 Cell Lines

Polysaccharides were rehydrated with autoclaved water to obtain working solutions at concentrations of 225 µg/mL for APPs and 200 µg/mL for DSPs to test their effect on A549 cell lines. The different working solution concentrations of APPs and DSPs were due to the availability of polysaccharides at the time of testing. However, the concentrations used were comparable and are, therefore, not expected to influence potential virucidal activity. To verify the possible toxic effect, an aliquot of each solution (75 µL) was added to wells containing 50 µL of A549 cell suspension and 75 µL of culture medium. In parallel, a control plate consisting of cells exposed to the virus but not treated with polysaccharides was used as the negative control of the experiment. After incubation at 37 °C and 5% CO_2_ for 24 h and 48 h, morphological alterations of the cells were evaluated by comparing treated and control wells using an inverted microscope.

Moreover, to evaluate whether exposure to polysaccharides affected cellular susceptibility to viral infection, A549 cells treated with non-cytotoxic concentrations of APPs or DSPs were subsequently infected with HAdV at the prepared viral titer (see Section 4.3). The presence of virus-induced cytopathic effect was then assessed as described below (Section 4.4).

### 4.6. Evaluation of APPs and DSPs Effect on HAdV5

To evaluate the virucidal effect of APPs and DSPs, the following experiments were conducted in triplicate for both viral titers. A total of 900 µL of the polysaccharide solution at the non-toxic concentration estimated (as reported in Section 4.5) was mixed with 100 µL of HAdV5 at two final concentrations, 5.65 × 10^3^ and 5.65 × 10^4^ TCID_50_/mL. The samples were incubated for 30 min. After that, 100 µL from each tube was transferred to an Eppendorf tube containing 900 µL of MEM. Then, three serial 1:10 dilutions of the mixture (sample + MEM) were prepared. This dilution step stopped the activity of the polysaccharides. A negative control (1 mL) was prepared using 900 µL of sterilized water and 100 µL of viral solution. Possible matrix interference was indirectly assessed through cytotoxicity experiments, in which polysaccharide solutions were incubated with A549 cells in MEM in the absence of virus (see Section 2.1). After 30 min of contact time, 100 µL from the samples and the negative control were taken, and viral titer was estimated as reported in Section 4.4. to assess viral abatement. To quantify viral infectivity, the TCID_50_/mL value was determined using the Spearman–Karber method as described above (see Section 4.4).

### 4.7. Evaluation of APPs Effect on HAdV5 at Different Contact Times

For APPs, to further study the virucidal effect, other experiments were performed in triplicate for three different contact times (15, 30 min and 60 min) at room temperature (23–25 °C). In each of them, a total of 1 mL suspension was prepared by mixing 900 µL of APPs, at the non-toxic concentration, and 100 µL of HAdV5, at the concentration of 4.24 × 10^3^ TCID_50_/mL. Negative control was prepared as described in Section 4.6. After each defined contact time, 100 µL from the samples and the negative control were taken, and viral titer was estimated to assess viral abatement.

### 4.8. Data Analysis

The viral abatement was expressed in terms of percentage and logarithmic (Log_10_) reductions. The percentage reduction (R%) was calculated using the formula R% = (1 − N_t_/N_0_) × 100, and the Log_10_ reduction (LR) was calculated as LR = Log_10_(N_0_/N_t_), where N_0_ is the viral titer at the beginning of each experiment, and N_t_ is the viral titer estimated after the different contact times (15, 30 and 60 min). For APPs, statistical analysis was performed using one-way ANOVA to compare viral titers, expressed as log_10_ TCID_50_/mL obtained after 15, 30, and 60 min of contact time. Results were considered significant with a *p*-value < 0.05. Statistical analyses were conducted using GraphPad Prism software (GraphPad, San Diego, CA, USA, version 5.03, 2009). Graphical representations of means, standard deviations, and viral reduction were created using Excel for Windows (Microsoft Office Excel, Redmond, WA, USA).

## 5. Conclusions

This study demonstrated the virucidal activity of polysaccharides extracted from cyanobacteria (*A. platensis*) and microalgae (*D. salina*) against human adenovirus, a highly resistant non-enveloped virus. However, the two polysaccharide preparations exhibited distinct behaviors: *A. platensis* polysaccharides, APPs, produced a measurable virucidal effect across the tested viral loads, whereas *D. salina* polysaccharides, DPSs, showed a viral load-dependent effect, with detectable reduction only at lower viral concentrations. These findings contribute to the current knowledge on the virucidal potential of algal polysaccharides against non-enveloped viruses. Nevertheless, as the present data were obtained under controlled in vitro conditions, further studies are required to assess their efficacy in more complex and realistic environmental scenarios, including the presence of interfering substances, organic load, variable pH, and different surface or water matrices. In addition, evaluating APPs and DSPs against other non-enveloped RNA viruses, such as poliovirus or norovirus, would help to better define the spectrum of virucidal activity and compare their effectiveness across different viral models. Finally, deeper structural characterization of the polysaccharide fractions and investigations of their mechanisms of action will be essential to clarify the determinants of the activity of sulfated polysaccharides obtained from *A. platensis* and *D. salina* and support future development.

## Figures and Tables

**Table 1:** Viral titration and abatement results of DSPs and APPs against HAdV5 at non-cytotoxic concentrations (DSPs 75.0 µg/mL, APPs 84.38 µg/mL) after 30 min of contact time with polysaccharides.

Spiking Virus	DSPs			APPs		
**Initial viral titer (TCID_50_/mL)**	Final viral titer (after 30 min contact time) (TCID_50_/mL) (GM ± sd)	Logarithmic reduction (mean ± sd)	Percentage reduction (%) (mean ± sd)	Final viral titer (after 30 min contact time) (TCID_50_/mL) (GM ± sd)	Logarithmic reduction (mean ± sd)	Percentage reduction (%) (mean ± sd)
**5.65 × 10^4^**	5.65 × 10^4^ ± 0.00	0.00 *	0.00 *	6.85 × 10^2^ ± 1.18	1.92 ± 0.07	98.78 ± 0.19
**5.65 × 10^3^**	4.24 × 10^2^ ± 0.00	1.13 ± 0.00	92.50 ± 0.00	6.85 × 10^1^ ± 1.18	1.92 ± 0.07	98.78 ± 0.19

GM = geometric mean; sd = standard deviation; * = no reduction detected under the experimental conditions.

**Table 2:** Viral titration and abatement results of APPs against HAdV5, at different contact times (15, 30 and 60 min).

Time	Viral Titer (TCID_50_/mL) (GM ± sd) *	Logarithmic Reduction (Mean ± sd)	Percentage Reduction (%) (Mean ± sd)
**15’**	6.85 × 10^1^ ± 2.30	1.16 ± 0.36	91.21 ± 6.44
**30’**	6.85 × 10^1^ ± 1.18	1.16 ± 0.07	93.09 ± 0.89
**60’**	4.67 × 10^1^ ± 1.18	1.33 ± 0.07	95.25 ± 0.66

GM = geometric mean; sd = standard deviation; * One-way ANOVA among contact times was not significant (*p* = 0.5822).

## Data Availability

The data that support the findings of this study are available from the corresponding author (A.P.) and M.V. upon reasonable request.

## Abbreviations

The following abbreviations are used in this manuscript:

Ad75	Adenovirus Type VI strain
AP	*Arthrospira platensis* Gomont
APPs	*Arthrospira platensis* polysaccharides
ATTC	American Type Culture Collection
CPE	Cytopathic Effect
DS	*Dunaliella salina*
DSPs	*Dunaliella salina* polysaccharides
HAdV	Human adenovirus type
HAdV5	Human adenovirus type 5
LR	Log_10_ Reduction
MEM	Minimal Essential Medium
MOI	Multiplicity of Infection
MTT assay	3-(4,5-dimethylthiazol-2-yl)-2,5-diphenyltetrazolium bromide assay
MWCO	Molecular Weight Cut-Offs
R%	Percentage Reduction
RSV	Respiratory Syncytial Virus
TCA	Trichloroacetic Acid
TCID_50_	50% Tissue Culture Infectious Dose

## References

[1] Reichling J. (2022). Antiviral and Virucidal Properties of Essential Oils and Isolated Compounds—A Scientific Approach. Planta Med..

[2] Rowe W.P., Huebner R.J., Gilmore L.K., Parrott R.H., Ward T.G. (1953). Isolation of a Cytopathogenic Agent from Human Adenoids Undergoing Spontaneous Degeneration in Tissue Culture. Exp. Biol. Med..

[3] Lion T. (2014). Adenovirus infections in immunocompetent and immunocompromised patients. Clin. Microbiol. Rev..

[4] Lynch J.P., Kajon A.E. (2016). Adenovirus: Epidemiology, Global Spread of Novel Serotypes, and Advances in Treatment and Prevention. Semin. Respir. Crit. Care Med..

[5] Takuissu G.R., Kenmoe S., Ebogo-Belobo J.T., Kengne-Ndé C., Mbaga D.S., Bowo-Ngandji A., Ondigui Ndzie J.L., Kenfack-Momo R., Tchatchouang S., Kenfack-Zanguim J. (2024). Exploring adenovirus in water environments: A systematic review and meta-analysis. Int. J. Environ. Health Res..

[6] Bofill-Mas S., Albinana-Gimenez N., Clemente-Casares P., Hundesa A., Rodriguez-Manzano J., Allard A., Calvo M., Girones R. (2006). Quantification and Stability of Human Adenoviruses and Polyomavirus JCPyV in Wastewater Matrices. Appl. Environ. Microbiol..

[7] Hewitt J., Greening G.E., Leonard M., Lewis G.D. (2013). Evaluation of human adenovirus and human polyomavirus as indicators of human sewage contamination in the aquatic environment. Water Res..

[8] Verani M., Federigi I., Donzelli G., Cioni L., Carducci A. (2019). Human adenoviruses as waterborne index pathogens and their use for Quantitative Microbial Risk Assessment. Sci. Total Environ..

[9] Federigi I., Salvadori R., Lauretani G., Leone A., Lippi S., Marvulli F., Pagani A., Verani M., Carducci A. (2024). Wastewater Treatment Plants Performance for Reuse: Evaluation of Bacterial and Viral Risks. Water.

[10] Thurston-Enriquez J.A., Haas C.N., Jacangelo J., Gerba C.P. (2003). Chlorine inactivation of adenovirus type 40 and feline calicivirus. Appl. Environ. Microbiol..

[11] Garcia L.A.T., Boff L., Barardi C.R.M., Nagl M. (2019). Inactivation of Adenovirus in Water by Natural and Synthetic Compounds. Food Environ. Virol..

[12] Verani M., Di Giuseppe G., Federigi I., Buonanno F., Ortenzi C., Carducci A. (2020). Preliminary Data Related to the Effect of Climacostol Produced by the Freshwater Ciliate Climacostomum virens on Human Adenovirus. Viruses.

[13] Musarra-Pizzo M., Pennisi R., Ben-Amor I., Mandalari G., Sciortino M.T. (2021). Antiviral Activity Exerted by Natural Products against Human Viruses. Viruses.

[14] García J.L., De Vicente M., Galán B. (2017). Microalgae, old sustainable food and fashion nutraceuticals. Microb. Biotechnol..

[15] Chaiklahan R., Chirasuwan N., Triratana P., Loha V., Tia S., Bunnag B. (2013). Polysaccharide extraction from *Spirulina sp*. and its antioxidant capacity. Int. J. Biol. Macromol..

[16] Borowitzka M.A. (1990). The mass culture of *Dunaliella salina*. Technical Resource Papers Regional Workshop on the Culture and Utilization of Seaweads.

[17] Mobin S., Alam F. (2017). Some promising microalgal species for commercial applications: A review. Energy Procedia.

[18] Chen Y.H., Wan X.Z., Wu D.S., Ouyang Y.Z., Gao L.Y., Chen Z.X., El-Seedi H.R., Wang M.F., Chen X.H., Zhao C. (2020). Characterization of the structure and analysis of the anti-oxidant effect of microalga *Spirulina platensis* polysaccharide on Caenorhabditis elegans mediated by modulating microRNAs and gut microbiota. Int. J. Biol. Macromol..

[19] Hou C., Chen L., Yang L., Ji X. (2020). An insight into anti-inflammatory effects of natural polysaccharides. Int. J. Biol. Macromol..

[20] Wang Q., Liu F., Chen X., Yang Z., Cao Y. (2020). Effects of the polysaccharide SPS-3-1 purified from *Spirulina* on barrier integrity and proliferation of Caco-2 cells. Int. J. Biol. Macromol..

[21] Wu X., Liu Z., Liu Y., Yang Y., Shi F., Cheong K.-L., Teng B. (2020). Immunostimulatory Effects of Polysaccharides from *Spirulina platensis* In Vivo and Vitro and Their Activation Mechanism on RAW246.7 Macrophages. Mar. Drugs.

[22] Goyal M., Baranwal M. (2017). Extracellular Polysaccharide Isolated from *Dunaliella salina* having Immunomodulatory and Cytotoxic Activity. Indo Glob. J. Pharm. Sci..

[23] Olasehinde T.A., Olaniran A.O., Okoh A.I. (2017). Therapeutic Potentials of Microalgae in the Treatment of Alzheimer’s Disease. Molecules.

[24] Goyal M., Baranwal M., Pandey S.K., Reddy M.S. (2019). Hetero-Polysaccharides Secreted from *Dunaliella salina* Exhibit Immunomodulatory Activity Against Peripheral Blood Mononuclear Cells and RAW 264.7 Macrophages. Indian J. Microbiol..

[25] Mishra A., Jha B. (2009). Isolation and characterization of extracellular polymeric substances from micro-algae *Dunaliella salina* under salt stress. Bioresour. Technol..

[26] Ilieva Y., Zaharieva M.M., Najdenski H., Kroumov A.D. (2024). Antimicrobial Activity of *Arthrospira* (Former *Spirulina*) and *Dunaliella* Related to Recognized Antimicrobial Bioactive Compounds. Int. J. Mol. Sci..

[27] Carlucci M.J., Scolaro L.A., Damonte E.B. (1999). Inhibitory action of natural carrageenans on Herpes simplex virus infection of mouse astrocytes. Chemotherapy.

[28] Kolender A.A., Pujol C.A., Damonte E.B., Matulewicz M.C., Cerezo A.S. (1997). The system of sulfated alpha-(1→3)-linked D-mannans from the red seaweed Nothogenia fastigiata: Structures, antiherpetic and anticoagulant properties. Carbohydr. Res..

[29] Talarico L.B., Zibetti R.G.M., Faria P.C.S., Scolaro L.A., Duarte M.E.R., Noseda M.D., Pujol C.A., Damonte E.B. (2004). Anti-herpes simplex virus activity of sulfated galactans from the red seaweeds *Gymnogongrus griffithsiae* and *Cryptonemia crenulata*. Int. J. Biol. Macromol..

[30] Thompson K.D., Dragar C. (2004). Antiviral activity of Undaria pinnatifida against herpes simplex virus. Phytother. Res..

[31] Hayashi T., Hayashi K., Maeda M., Kojima I. (1996). Calcium Spirulan, an Inhibitor of Enveloped Virus Replication, from a Blue-Green Alga *Spirulina platensis*. J. Nat. Prod..

[32] Damonte E.B., Matulewicz M.C., Cerezo A.S. (2004). Sulfated seaweed polysaccharides as antiviral agents. Curr. Med. Chem..

[33] Harden E.A., Falshaw R., Carnachan S.M., Kern E.R., Prichard M.N. (2009). Virucidal activity of polysaccharide extracts from four algal species against herpes simplex virus. Antivir. Res..

[34] Marchetti M., Pisani S., Pietropaolo V., Seganti L., Nicoletti R., Orsi N. (1995). Inhibition of herpes simplex virus infection by negatively charged and neutral carbohydrate polymers. J. Chemother..

[35] Polini B., Banti M., Mazzierli A., Corti A., Nieri P., Manera C., Chiellini G. (2026). Sulfated Polysaccharide-Rich Fractions from *Spirulina Platensis* (SPPs) Exert Multi-Target Anticancer Activity in Non-Small Cell Lung Cancer (NSCLC) Cells. Pharmaceuticals.

[36] Hans N., Malik A., Naik S. (2021). Antiviral activity of sulfated polysaccharides from marine algae and its application in combating COVID-19: Mini review. Bioresour. Technol. Rep..

[37] Liyanage N.M., Nagahawatta D.P., Jayawardena T.U., Sanjeewa K.K.A., Jayawrdhana H.H.A.C.K., Kim J.-I., Jeon Y.-J. (2023). Sulfated Polysaccharides from Seaweeds: A Promising Strategy for Combatting Viral Diseases—A Review. Mar. Drugs.

[38] Banti M., Garcia-Gil M., Guidotti L., Di Giuseppe G., Rapposelli S., Monti D., Tampucci S., De Leo M., Gado F., Nieri P. (2025). Characterization and Otoprotective Effects of Polysaccharides from *Arthrospira platensis*. Molecules.

[39] Chen Y.-H., Chang G.-K., Kuo S.-M., Huang S.-Y., Hu I.-C., Lo Y.-L., Shih S.-R. (2016). Well-tolerated *Spirulina* extract inhibits influenza virus replication and reduces virus-induced mortality. Sci. Rep..

[40] Chen W., Chen Y.-H., Liao Y.-C., Huang X.-W., Lu T.-J., Shih S.-R. (2023). Effect of hot water extracts of *Arthrospira maxima* (spirulina) against respiratory syncytial virus. Phytomedicine.

[41] El-Baz F.K., El-Senousy W.M., El-Sayed A.B., Kamel M.M. (2013). In vitro antiviral and antimicrobial activities of *Spirulina platensis* extract. J. Appl. Pharm. Sci..

[42] Mader J., Gallo A., Schommartz T., Handke W., Nagel C.H., Günther P., Brune W., Reich K. (2016). Calcium spirulan derived from *Spirulina platensis* inhibits herpes simplex virus 1 attachment to human keratinocytes and protects against herpes labialis. J. Allergy Clin. Immunol..

[43] Ohta S., Ono F., Shiomi Y., Nakao T., Aozasa O., Nagate T., Kitamura K., Yamaguchi S., Nishi M., Miyata H. (1998). Anti-Herpes Simplex Virus substances produced by the marine green alga, *Dunaliella primolecta*. J. Appl. Phycol..

[44] Singab A.N., Ibrahim N., Elsayed A.E., El-Senousy W., Aly H., Abd Elsamiae A., Matloub A. (2018). Antiviral, cytotoxic, antioxidant and anti-cholinesterase activities of polysaccharides isolated from microalgae *Spirulina platensis*, *Scenedesmus obliquus* and *Dunaliella salina*. Arch. Pharm. Sci. Ain Shams Univ..

[45] (2025). Chemical Disinfectants and Antiseptics—Quantitative Suspension Test for the Evaluation of Virucidal Activity in the Medical Area—Test Method and Requirements (Phase 2/Step 1).

[46] Jiao G., Yu G., Zhang J., Ewart H.S. (2011). Chemical structures and bioactivities of sulfated polysaccharides from marine algae. Mar. Drugs.

[47] Dong X., Qiu Y., Jia N., Wu Y., Nie Q., Wen J., Zhao C., Zhai Y. (2025). Recent advances of edible marine algae-derived sulfated polysaccharides in antiviral treatments: Challenges vs. opportunities. Front. Nutr..

[48] Walne P.R. (1996). Experiments in the Large-Scale Culture of Larvae of Ostrea edulis L..

[49] Chen W., Gao L., Song L., Sommerfeld M., Hu Q. (2023). An improved phenol-sulfuric acid method for the quantitative measurement of total carbohydrates in algal biomass. Algal Res..

[50] Carducci A., Federigi I., Balestri E., Lardicci C., Castelli A., Maltagliati F., Zhao H., Menicagli V., Valente R., De Battisti D. (2022). Virus contamination and infectivity in beach environment: Focus on sand and stranded material. Mar. Pollut. Bull..

[51] Hierholzer J.C., Killington R.A., Mahy B.W.J., Kangro H.O. (1996). Virus isolation and quantitation. Virology Methods Manual.

[52] Ramakrishnan M.A. (2016). Determination of 50% endpoint titer using a simple formula. World J. Virol..

